# How should the electrosurgical mode be optimized for endoscopic sphincterotomy?

**DOI:** 10.1055/a-2466-8038

**Published:** 2024-11-28

**Authors:** Horst Neuhaus

**Affiliations:** 1 Gastroenterology, Interdisciplinary Care Clinic RKM740, Düsseldorf, Germany

**Keywords:** Pancreatobiliary (ERCP/PTCD), Stones, Strictures, ERC topics



Endoscopic sphincterotomy (EST) of the biliary sphincter is a well-established procedure for treating disorders of the papilla of Vater and facilitating interventional endoscopic retrograde cholangiopancreatography (ERCP)-guided procedures. Adverse events (AEs) of EST occur in up to 10% of cases and are mainly related to bleeding, post-ERCP pancreatitis (PEP), cholangitis, and rarely perforation. PEP also can be caused by difficult cannulation prior to EST. Risk factors have been identified for each of these AEs and they should be considered when selecting patients and interpreting results of EST studies. Several guidelines recommend various measures to minimize risk of PEP. These include routine rectal administration of nonsteroidal anti-inflammatory drugs (NSAIDs) before ERCP in all patients without contraindications, which leads to a 50% reduction in PEP. In addition, pancreatic stents should be used in high-risk patients in whom the pancreatic duct has been repeatedly or deeply accessed 1 2 .

Choice of electrosurgical current for EST remains a source of controversy. The European Society of Gastrointestinal Endoscopy (ESGE) recommends, based on moderate-level evidence, that mixed current should be applied for EST rather than pure cut current alone due to a decreased risk of mild bleeding. A mode providing alternating cutting and coagulation phases (e.g. endoCUT I; hereafter referred to as Endocut) is suggested instead of conventional blended current because it may be associated with a lower risk of uncontrolled cutting and intraprocedural bleeding 3 . A recent network meta-analysis included nine randomized controlled trials (RCTs) with a total of 1615 patients comparing four electrocautery modes (blended cut, pure cut, Endocut and pure cut followed by blended cut) for EST 4 . The results showed that no electrocautery mode was superior to the others in preventing PEP; nevertheless, Endocut was found to be superior to the others with respect to preventing bleeding. Therefore, the authors suggest performing EST with Endocut in accordance with the ESGE guidelines.

The newest technology of Endocut allows operators to choose between two cutting modes and to modify the level of coagulation (effect), incision time (cutting duration), and the cycle of incision time and incision pause time during which coagulation is applied (interval). An automatic electric arc (spark) detection ensures a reproducible and controlled cutting result and the system offers 320 different setting options. endoCUT I mode with levels 2 for effect, 2 for duration, and 2 for interval (2–2-2) seems to be an appropriate choice for the majority of cases of biliary EST. However, the level of evidence is low due to a lack of appropriate clinical trials, which are difficult to perform because of the high number of influencing factors and variables 5 .

Therefore, the report by De Oliveira et al. in a recent issue of Endoscopy International Open 6 has been read with great interest. Their systematic review and meta-analysis included 987 patients from four RCTs comparing Endocut with pure cut for EST. The results showed a significantly higher risk of PEP in the Endocut group than in the pure cut group. On the other hand, Endocut resulted in a significantly lower incidence of intraprocedural bleeding than pure cut (10% vs 19%, *P* = 0.05). However, all EST-related bleeding episodes could be managed during the procedures and had no clinical repercussions. Incidence of delayed bleeding was comparable for the techniques. From these findings, the authors suggest that pure cut should be the preferred electrosurgical mode for biliary EST.

Should the current guidelines be amended accordingly? This does not seem justified, given several limitations of the meta-analysis, which are only partially discussed by the authors 6 . They included four studies with a dominance of one trial that enrolled more than half of all patients (550/987) 7 . This selection may lead to bias, especially because two of the three other studies were only published as congress abstracts. All authors of the meta-analysis are from the same institutions as the authors of the dominant study, resulting in a potential conflict of interest.

Rectal NSAIDs were not administered, and pancreatic stents in high-risk patients were not used in any of these studies, which represents a deviation from standard care guidelines for prevention of PEP. Therefore, the meta-analysis unfortunately cannot provide information about the impact of electrocautery modes on risk of PEP when EST is performed according to current standards.

Further limitations of the meta-analysis are related to a lack of standardization of electrosurgical devices. Two studies did not provide any information about settings of modes or even about the type of generator. Standardization was partially present in the dominant study by Funari et al. 7 because four different electrosurgical devices were used during the study across two separate study centers; however, the application of Endocut was conducted with fixed settings for the effect, cutting duration, and cutting interval. This protocol is appropriate for the purpose of a study, but results can only be interpreted with regard to the specific setting. It precludes taking advantage of the latest generators to adjust Endocut settings depending on individual factors and procedure variants such as the size of the contact surface between the cutting wire and tissue 5 . For patients with risk factors for PEP or those with a small papilla, for example, the effect can be reduced and/or the cutting interval shortened to minimize coagulation without sacrificing the benefits of alternating phases.

EST with pure cut caused intraprocedural bleeding in every fifth case. The authors of the dominant study emphasize that all cases were endoscopically controlled with “relatively simple measures” 7 . However, it should be considered that all procedures were performed in high-volume centers with extensive experience in endoscopic hemostasis. Less skilled and experienced endoscopists may experience greater problems and feel stressed. In addition, endoscopic management of bleeding can be time-consuming and increase costs because of the use of additional devices. The meta-analysis did not show a significant difference between the groups in risk of a fast, uncontrolled, large cut event ("zipper" cut). There is no clear definition for this event, but in one of the trials, it was significantly more frequently observed after pure cut compared with Endocut (8% vs. 0%, *P* = 0.02) 8 . Endoscopists with limited experience in EST, in particular, may appreciate the controlled stepwise cutting with Endocut.

The follow-up protocols for the two fully-reported trials in the meta-analysis included laboratory tests on the first postprocedure day in one study and patient interviews after 7 days in both trials 7 8 . Therefore, the results regarding delayed AEs showing no differences between the groups should be interpreted cautiously.

## Conclusions

In conclusion, the meta-analysis provides valuable insights but is influenced by methodological shortcomings and the dominance of the study by Funari et al. 7 . A sensitivity analysis excluding this study would most probably reveal the potential for different conclusions and highlights the need for cautious interpretation of the results. The reported data do not justify changes to the current guidelines. They suggest minimizing the effect of coagulation for EST, especially in patients with increased risks for PEP. This can be easily achieved with Endocut, e.g., by changing settings to 1–1-1 without sacrificing the advantages of this current mode. Modern electrosurgical generators allow operators to individualize settings depending on various characteristics. The prerequisite is that endoscopists are familiar with the technology and its effects on tissue. It is unlikely and may not make sense to define a single optimal setting for all cases, just as no single optimal setting exists for other endoscopic interventions such as endoscopic submucosal dissection. Further research should consider an individualized approach. It requires transparent disclosure of potential biases, inclusion of diverse data sources, and adherence to rigorous methodological standards to provide robust and reliable clinical recommendations.
